# BMC Palliative Care reviewer acknowledgement, 2012

**DOI:** 10.1186/1472-684X-12-16

**Published:** 2013-04-25

**Authors:** Hayley Henderson

**Affiliations:** 1 BioMed Central, Floor 6, 236 Gray's Inn Road, London WC1X 8HB, United Kingdom

## Abstract

**Contributing reviewers:**

The editors of *BMC Palliative Care* would like to thank all our reviewers who have contributed to the journal in Volume 11 (2012).

## 

Simon Allan

New Zealand

Alan Astrow

USA

Sabine Bader

Switzerland

Stephen Barclay

United Kingdom

Claudia Bausewein

United Kingdom

Gerhild Becker

Germany

Christina Bell

USA

Antonia Beringer

United Kingdom

Teresa Beynon

United Kingdom

Lauren Breen

Australia

Karen Brombley

United Kingdom

Hilde Buiting

Netherlands

Marylou Cardenas-Turanzas

USA

Lindsay Carey

Australia

Colleen Cartwright

Australia

Jack Chen

USA

Joachim Cohen

Belgium

Eileen Cowey

United Kingdom

Isolde Daiski

Canada

Ruth Davies

United Kingdom

Mamede De Carvalho

Portugal

Maxine De La Cruz

USA

Julia Downing

Uganda

Annette Edwards

United Kingdom

Maria Friedrichsen

Sweden

Jan Gaertner

Germany

Paul H Gordon

France

Gunn Grande

United Kingdom

Michael Harlos

Canada

Pippa Hawley

Canada

Marilynne Hebert

Canada

Paul Howard

United Kingdom

David Hui

USA

Christine Ingleton

United Kingdom

Akira Inoue

Japan

Barbara Jack

United Kingdom

Donna Johnston

Canada

Jung Hye Kwon

Korea, South

Richard Latten

United Kingdom

Wendy Lichtenthal

USA

Christopher J. Mackinnon

Canada

Juan Carlos Martinez Castrillo

Spain

Susan Mcclement

Canada

Anne Merriman

Uganda

Tatsuya Morita

Japan

Faith Mwangi-Powell

Uganda

Cheryl Nekolaichuk

Canada

David Ngo

United Kingdom

Bregje D Onwuteaka-Philipsen

Netherlands

H. Roeline W. Pasman

Netherlands

Sophie Pautex

Switzerland

Carina Persson

Sweden

Karen Petchey

United Kingdom

Jane Phillips

Australia

Aron Frederik Popov

Germany

Holly Prigerson

USA

Saifudin Rashiq

Canada

Edward Ratner

USA

John Rosenberg

Australia

Jayesh Sagar

United Kingdom

Christian Schulz

Germany

Lucy Selman

United Kingdom

Jane Seymour

United Kingdom

Cathy Shipman

United Kingdom

Imke Strohscheer

Germany

Jeanine Suurmond

Netherlands

Keith Swetz

USA

Heather Tan

Australia

Sally Thorne

Canada

Lieve Van Den Block

Belgium

Karin Van Der Rijt

Netherlands

Bee Wee

United Kingdom

David Widdas

United Kingdom

David Wright

Canada

Sriram Yennu

USA

## Pre-publication history

The pre-publication history for this paper can be accessed here:

http://www.biomedcentral.com/1472-684X/12/16/prepub

